# The reproductive stress hypothesis

**DOI:** 10.1530/REP-18-0592

**Published:** 2019-07-23

**Authors:** Lixin Wen, Rongfang Li, Ji Wang, Jine Yi

**Affiliations:** 1 Department of Clinical Veterinary Medicine, College of Veterinary Medicine, Hunan Agricultural University, Changsha, People’s Republic of China; 2 Hunan Collaborative Innovation Center of Animal Production Safety, Changsha, People’s Republic of China

## Abstract

In this paper, we propose the reproductive stress hypothesis that describes the pregnant females response to reproductive events based upon the activation of the hypothalamic–pituitary–adrenal axis and sympathetic adrenomedullary system. The main components of the reproductive stress hypothesis can be summarized as follows: (1) events unique to reproduction including empathema, pregnancy, parturition and lactation cause non-specific responses in females, called active reproductive stress; (2) the fetus is a special stressor for pregnant females where endocrine hormones, including corticotropin-releasing hormones and fetal glucocorticoids secreted by the fetus and placenta, enter the maternal circulatory system, leading to another stress response referred to as passive reproductive stress and (3) response to uterine tension and intrauterine infection is the third type of stress, called fetal intrauterine stress. Appropriate reproductive stress is a crucial prerequisite in normal reproductive processes. By contrast, excessive or inappropriate reproductive stress may result in dysfunctions of the reproductive system, such as compromised immune function, leading to susceptibility to disease. The novel insights of the reproductive stress hypothesis have important implications for deciphering the pathogenesis of certain diseases in pregnant animals, including humans, which in turn may be applied to preventing and treating their occurrence.

## Introduction

The concept of stress was first introduced to the field of medicine and biology by the pioneer Hans Selye in 1936, where it was defined as ‘the non-specific response of the body to any demand’. Stress is a bodily, psychological or emotional factor that causes physical or mental tension. Any stimulation including external (environmental, psychological or social) or internal (disease or medical procedures) can induce stress. During times of heightened stress, the body activates the hypothalamic–pituitary–adrenal (HPA) axis (Smith & Vale 2006) and sympathetic adrenomedullary system (SAS) (Carter *et al.* 2015) in response to either real or perceived threats. This results in a cascade of hormone releases including adrenocorticotropic hormone (ACTH), corticotropin-releasing hormone (CRH), cortisol, epinephrine (E) and norepinephrine (NE) (Chrousos 2000). Once the stress response is activated, behavioral and physiological changes adjust homeostasis to increase the chances of survival (Van de Kar & Blair 1999). In general, reproduction is a physiological process in mammals and regarded as a special stressor. At the symposium on the Internal Medicine of Domestic Animals of the Chinese Association of Animal Husbandry and Veterinary Medicine in 2006, we put forward the first iteration of a reproductive cycle hypothesis. For over 10 years, we have supplemented and improved this hypothesis and its application which are reviewed herein.

## Theoretical basis of the reproductive stress hypothesis

**Figure 1:**
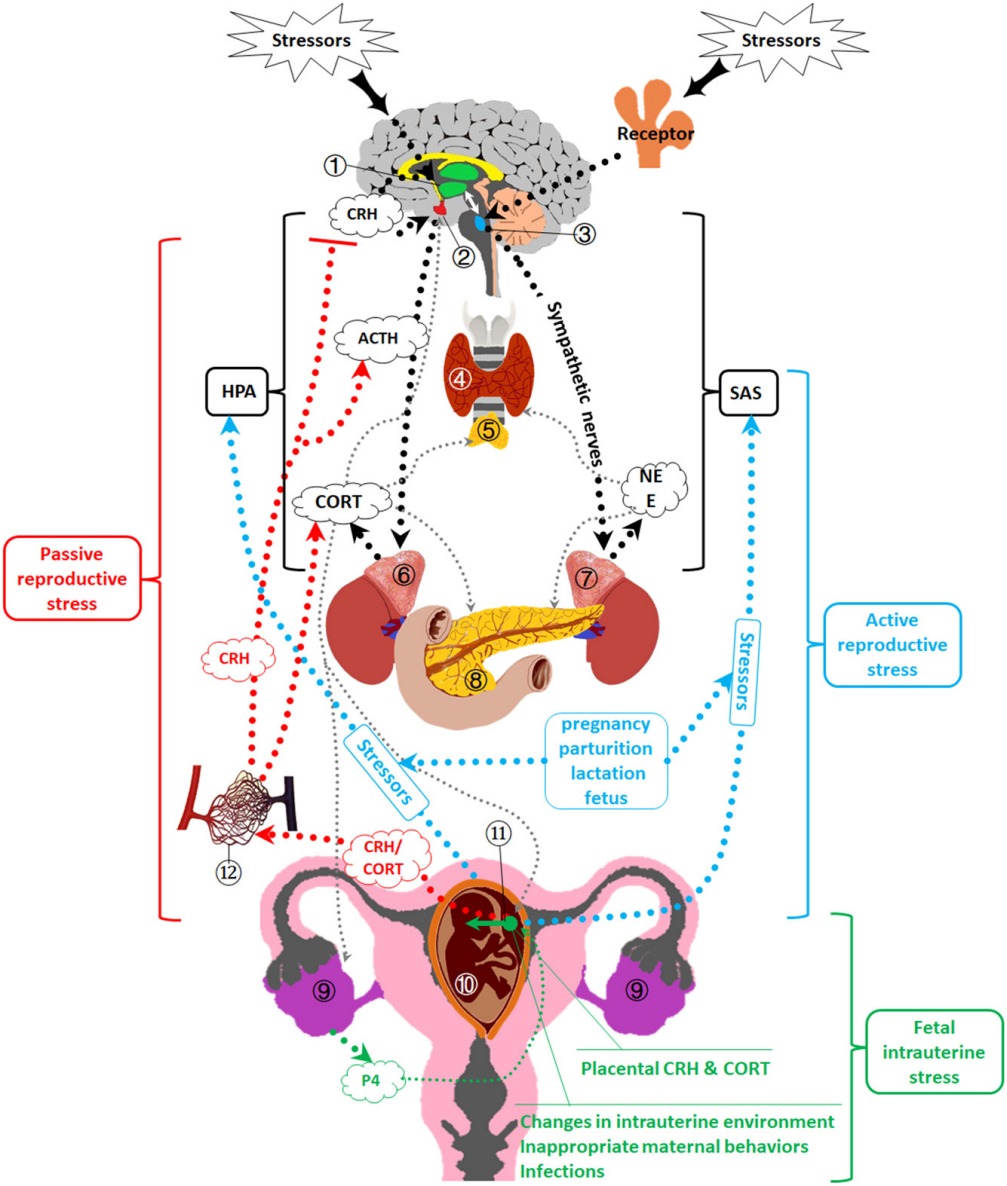
Mechanisms of regular stress and reproductive stress. ① Hypothalamus; ② pituitary; ③ locus coeruleus; ④ thyroid; ⑤ thymus; ⑥ adrenal cortex; ⑦ adrenal medulla; ⑧ islet; ⑨ ovary; ⑩ fetus; ⑪ placental barrier and ⑫ maternal circulatory system. Regular stress includes hypothalamic–pituitary–adrenal (HPA) axis and sympathetic adrenomedullary system (SAS), as illustrated with black symbols. Reproductive stress includes active reproductive stress, passive reproductive stress and fetal intrauterine stress. Active reproductive stress: the fetus and the processes of pregnancy, parturition and lactation act as stressors, triggering the HPA axis and the SAS, as illustrated with blue symbols. Passive reproductive stress: fetal and placental corticotropin-releasing hormone (CRH) and corticosterone (CORT) are secreted into the maternal circulatory system, the CRH inhibits the hypothalamus-secreted CRH and stimulates the secretion of adrenocorticotropic hormone (ACTH). The free CORT immediately acts on target organs through the maternal circulatory system, as illustrated with red symbols. Fetal intrauterine stress: during pregnancy, the placental CRH/CORT, infections, changes in intrauterine environment, as well as inappropriate maternal behaviors are stressors for the fetus, as illustrated with green symbols.

As a Chinese saying goes, ‘It is a matter of life or death when a mother faces delivering a baby after ten months of pregnancy’. Indeed, the reproduction process is life or death for the mother. Reproduction is a special stressor that overcomes the effect of routine external forces, activating a stress response that we refer to as reproductive stress. Herein, we define reproductive stress as the non-specific response of the body to reproductive activities including the estrous cycle, pregnancy, parturition and lactation. According to our hypothesis, reproductive stress includes active reproductive stress, passive reproductive stress and fetal intrauterine stress (Fig. 1).

### Active reproductive stress

The reproductive stress that activates the maternal HPA axis and SAS is designated as active reproductive stress in our hypothesis. Basal HPA activity varies with the fluctuation of the estrous cycle (Viau & Meaney 1991, Atkinson & Waddell 1997) and is highest at the end of the follicular phase or at the beginning of the luteal phase in human beings (Altemus *et al.* 2001). This is because both ACTH and cortisol as well as hypothalamic CRH content are increasing (Atkinson & Waddell 1997). Additionally, during proestrus, catecholamines increase rapidly before ovulation. This suggests that estrous can be a stressor. The enhanced HPA activity during proestrus may serve as a protective mechanism for successful reproductive activity during estrous phases (Viau & Meaney 1991).

To provide an appropriate intrauterine environment for the developing fetus, the maternal HPA system undergoes crucial alterations (Oyola & Handa 2017, Gilles *et al.* 2018). Although some studies have reported a plateau in total plasma cortisol concentrations and alleviation in HPA axis stress responses during late pregnancy (Russell *et al.* 2008), other studies have demonstrated that the HPA axis and SAS are continually activated during pregnancy and parturition (Jung *et al.* 2011, Gilles *et al.* 2018). The maternal serum cortisol concentrations increase to 3-times the nonpregnant level throughout pregnancy in parallel to the rise in plasma corticosteroid-binding globulin (CBG) (Jung *et al.* 2011). The elevation of glucocorticoids and CRH in maternal and fetal plasma is closely associated with parturition (McLean *et al.* 1995), an indicator that parturition is an important event that leads to reproductive stress in pregnant females. Moreover, plasma levels of several components of the rennin-angiotensin-aldosterone system are increased during normal pregnancy (August *et al.* 1995). Notably, placental production of CRH, which is stress sensitive (Christian 2012), can influence the timing of parturition, namely preterm, normal term or post-term labor (McLean *et al.* 1995, Mastorakos & Ilias 2000, Hobel *et al.* 2008).

In formulating the reproductive stress hypothesis, we consulted a large body of work to compare changes in maternal hormones related to general stress and reproductive stress (mainly pregnancy and childbirth) (Table 1). The results show that the changes in stress hormone levels during reproductive stress are consistent with those during general stress, where the levels of E, NE, CRH, ACTH and glucocorticoids in plasma are increased. The changes in other major hormones, inflammatory mediators and C-reactive proteins in the body are also similar. However, reproductive stress is different from general stress when considering certain hormones. For example, levels of both insulin and glucagon rise in late gestation (Saudek *et al.* 1975), but a reduction of insulin sensitivity is observed in normal pregnancy (Valsamakis *et al.* 2017).

**Table 1:** Comparison of predominant hormones between regular stress and reproductive stress.

Hormones	Regular stress	Pregnancy or parturition	References
E/NE	Increase	Increase	Hydbring *et al*. 1999, Chrousos 2000, Gilles *et al.* 2018
CRH-ACTH-Glucocorticoid	Increase	Increase	McLean & Smith 1999, Mastorakos & Ilias 2003, Jung *et al.* 2011, Valsamakis *et al.* 2019
Renin-Angiotensin-Aldosterone	Increase	Increase	August *et al.* 1995, Lumbers & Pringle 2014, Verdonk *et al.* 2014
Dopamine	Increase	Increase	Ben-Jonathan & Maxson 1978, Tombeau Cost *et al.* 2017
Prolactin	Increase	Increase	Baan *et al*. 2008, Wagenmaker & Moenter 2017
Prostaglandin	Increase	Increase	Challis *et al*. 1997, Wagenmaker & Moenter 2017
Glucagon	Increase	Increase	Saudek *et al.* 1975, Harp *et al.* 2016
Insulin	Decrease	Increase with insulin resistance	Saudek *et al.* 1975, Kamba *et al*. 2016, Valsamakis *et al.* 2017
Testosterone	Decrease	Decrease	Carlsen *et al*. 2003, Oyola & Handa 2017
LH	Decrease	Decrease	Baan *et al*. 2008, Wagenmaker & Moenter 2017
GnRH	Decrease	Decrease	Sorem *et al*. 1996, Raftogianni *et al.* 2018
Progestation	Decrease	Decrease or functional withdraw	Mitchell & Taggart 2009, Oyola & Handa 2017

ACTH, adrenocorticotropic hormone; CRH, corticotropin-releasing hormone; E, epinephrine; GnRH, gonadotropin-releasing; NE, norepinephrine.

This accumulating evidence indicates that reproductive activities, especially estrus, pregnancy and parturition are important stressors that can lead to a state of active reproductive stress.

### Passive reproductive stress

The fetoplacental unit is an essential, special stressor that causes the secretion of endocrine hormones including CRH and glucocorticoids. During gestation, CRH production in the placenta (mainly in humans and other anthropoid primates), decidua and fetal membranes are responsible for the dramatic rise in circulating maternal immunoreactive CRH from the first trimester of pregnancy (Mastorakos & Ilias 2003), keeping pace with the maturing fetus during the second and third trimesters. In addition to the maternal sources, the mature fetus can also produce CRH, which causes the maternal CRH to peak during labor (Chen *et al.* 2010). The responsiveness of the mother to the fetus is termed passive reproductive stress in our hypothesis. It is distinct from active reproductive stress that involves maternal activation of the HPA axis and SAS. CRH acts on the fetal pituitary through CRH receptors, which can activate the fetal HPA axis. This leads to the release of ACTH, which acts on fetal adrenal glands and produces large amounts of cortisol and DHEAS (Ravanos *et al.* 2015). High cortisol concentration promotes fetal lung maturation and gene expression of CRH, prostaglandin and oxytocin in the placenta. Moreover, fetal CRH is regulated by fetal CRH-binding protein (CRH-BP) and glucocorticoids. CRH-BP can prevent placental CRH from overstimulating the pituitary gland, which may be one of the reasons for the mild elevation of ACTH levels in maternal blood (Thomson 1998).

### Fetal intrauterine stress

Within the uterus, the fetus is vulnerable and exposed to many forms of stressors, including uterine tension, oligohydramnios, and potential intrauterine infection derived from disease, smoking or alcohol in the bloodstream. The responsiveness of the fetus to these stressors is called fetal intrauterine stress. Activation of the HPA axis is the main adaptive response of fetal intrauterine stress (Maršál 2018). However, the feedback of CRH by cortisol in intrauterine tissue is significantly different from that of the adult HPA axis (Menon *et al.* 2016). The former is a positive feedback loop, which produces exponential glucocorticoids before parturition, synchronizing with delivery and fetal organ maturation. However, premature birth occurs if the intrauterine environment is too extreme for fetal survival (McLean & Smith 1999).

The sequence of events between mother and fetus that trigger parturition in humans has not been fully elucidated. The promulgated theories include fetal membrane senescence (Menon *et al.* 2016), progesterone withdrawal, maternal CRH release, fetal HPA axis activation, inflammatory and mechanical factors (Ravanos *et al.* 2015). Our hypothesis posits that fetal intrauterine stress is the prime instigator of normal labor. According to the hypothesis, it is therefore predictable that multiple gestations will result in premature birth with the risk in triplets higher than for twins. Indeed, numerous reports confirm this response. In pregnant marmoset models, scientists have showed that maternal peak and mean values for circulating CRH are correlated with fetal number. For example, pregnant females with triplets have higher CRH values than those with twins and those carrying a single fetus have the lowest value (Power *et al.* 2006). At present, premature birth risk is predicted by measuring plasma CRH concentrations. An overwhelming amount of data have demonstrated that the preterm delivery rate for multiple gestations is several times higher than for singletons (Heino *et al.* 2016, Vogel *et al.* 2018). One-third of triplets are delivered before 32 weeks of pregnancy (Papageorghiou *et al.* 2006). One study reported that the risk of preterm birth was nine-fold higher in multiple births than in singletons (Heino *et al.* 2016). An analysis of national data from 19 European countries (2004–2008) found that the increased prematurity rates in most countries was driven by higher rates of multiple births, as well as higher rates of prematurity among multiple births (Zeitlin *et al.* 2013). In some instances, these risks have resulted in selective reduction, called multifetal pregnancy reduction (from three to two fetuses in the case of triplets), in order to reduce the risk of severe preterm birth and high risk of miscarriage (Papageorghiou *et al.* 2006). In addition, the timing of parturition was significantly disrupted in experimental anencephaly (functional hypophysectomy) of the rhesus fetus, resulting in 30% preterm and 40% post-term delivery (Novy 1977), which strongly supports the importance of fetal intrauterine stress on initiation of parturition. In addition, fetal adrenal glands are large relative to adult adrenals (Mastorakos & Ilias 2003, Bronstein *et al.* 2015), providing a powerful engine for childbirth.

### Physiological consequences of reproductive stress

Obviously, reproductive stress plays a significant role in human and animal reproduction processes. It exerts a wide array of physiological consequences in maternal hematologic, metabolic, endocrine and immune systems and plays a key role in events underlying fetal organ development. Maintenance of elevated serum aldosterone and cortisol concentrations during pregnancy is essential for the normal gestational increase in uterine blood flow (Jensen *et al.* 2005, Charkoudian *et al.* 2017). Physiologically, plasma volume increases by 10–15% at 6–12 weeks of gestation and then increases rapidly until 30–34 weeks (Whittaker *et al.* 1996, Jwa *et al.* 2015). It is believed that this is an adaptive mechanism to expand the plasma volume allowing for appropriate utero-placental perfusion and indirectly supports fetal arterial oxygen tension, blood pressure and development of the HPA axis (Jensen *et al.* 2002). Thus, reduction in maternal aldosterone and cortisol levels can disrupt both maternal and fetal homeostasis (Jensen *et al.* 2002). This may result in a compromised maternal state of intrauterine growth restriction (West *et al.* 2014) and contribute to premature birth and neonatal morbidity (Schneiderman *et al.* 2017, Brue *et al.* 2018). Glucocorticoids are the determinants of cell proliferation inhibition, terminal differentiation stimulation and a basic switch for fetal organ maturation (Miranda & Sousa 2018). The most well-known function is to stimulate differentiation and functional development of the lungs (Busada & Cidlowski 2017), the primary adaptative feature of aerobic life (Wood & Walker 2015). Mothers showing signs of premature labor are widely administered antenatal glucocorticoids to accelerate fetal lung development and prevent respiratory distress syndrome in preterm infants (Brownfoot *et al.* 2013, Busada & Cidlowski 2017). Historically, maternal tolerance of a semiallogeneic fetus was thought to be achieved through immunosuppression (Racicot *et al.* 2014). It is now well recognized that the maternal immune system not only adapts during pregnancy, but also actively participates in all stages of the reproductive process. While glucocorticoids have been suggested to regulate reproductive function through gonadotropin (Gore *et al.* 2006) and glucocorticoid receptors (GRs) (Whirledge *et al.* 2015), recent studies have shown that female mice lacking GRs specifically in the uterus are subfertile, exhibiting defects in embryo implantation and subsequent decidualization related to inadequate remodeling of the endometrial stroma (Whirledge *et al.* 2015). In addition to their effect on lung development and the reproductive system, glucocorticoids play an essential role in accelerating the development of several other organ systems, such as the fetal cardiovascular system (Unno *et al.* 1999, Rog-Zielinska *et al.* 2014), gastrointestinal tract (Lebenthal *et al.* 1999), liver (Fowden *et al.* 2011) and brain (Buss *et al.* 2012) for the transition to extrauterine life. Late fetal glucocorticoids also regulate metabolic functions, including thyroid hormone secretion, glycometabolic enzyme activity in the liver and fat reserve mobilization for the brain and liver during the first postnatal days (Wood & Walker 2015).

## Reproductive stress syndrome

However, reproductive stress is a sustained and prolonged process for pregnant females, potentially resulting in alteration of neuroendocrine events, changes in metabolism (Parisi *et al.* 2019), impairment of immune function and possible dysfunction of the reproductive system, which we designate as reproductive stress syndrome. Its clinical symptoms are complex and particular to reproductive activities but can be summarized into three categories.

First, an important clinical manifestation of reproductive stress syndrome is the abnormality of bone metabolism, which may result in osteoporosis over time. This can happen when maternal nutrition supply is insufficient or reproductive tasks are aggravated, especially during the third trimester when the rate of fetal bone accretion is increasing. The maternal stress mechanism mobilizes the body to store nutrients to ensure the needs of offspring (Hacker *et al.* 2012). To ensure fetal growth and lactation, mobilization of body reserves is needed to accelerate the loss of nutrients such as calcium (Eisman 1998, Kovacs & Ralston 2015). On the other hand, excess glucocorticoids have harmful effects on the proliferation and differentiation of osteoblasts as well as the survival of osteoblasts and osteocytes, leading to the acceleration of the apoptosis and/or autophagy of osteoblasts (Komori 2016). Meanwhile, large amounts of endogenous glucocorticoids decrease bone strength via interconnected decrements in bone angiogenesis, vasculature volume and osteocyte–lacunar–canalicular fluid in osteoblasts and osteocytes (Weinstein *et al.* 2010).

A second clinical symptom of reproductive stress syndrome is reproductive disorders including sexual dysfunction in postpartum women (Khajehei *et al.* 2015) and the delay or absence of estrus. During the prepartum period, the reproductive stress mechanism is characterized by HPA axis activation and hypothalamic–pituitary–gonadal axis suppression (Mitchell *et al.* 2005, Grachev *et al.* 2014). High levels of CRH or arginine vasopressin secreted by the hypothalamus suppresses gonadotropin-releasing hormone and/or lutenizing hormone secretion (Dobson *et al.* 2003, Ciechanowska *et al.* 2018). Moreover, undernutrition or a large amount of body reserve loss results in the body’s negative energy balance, which induces persistently low levels of insulin (Lucy 2008) and decreases the expression of growth hormone receptors in the liver and the secretion of insulin-like growth factor-1 (IGF-1). This negatively impacts reproduction as insulin and IGF-1 are unable to synergize with the gonadotrophins on ovarian cells, preventing the dominant follicle from ovulating and delaying the resumption of the estrous cycle, thereby inhibiting reproductive function (Walsh *et al.* 2011).

A third clinical aspect of reproductive stress syndrome is the potential decrease in immune function resulting in increased disease during pregnancy and parturition. Pregnancy and the postpartum period are marked by high glucocorticoids levels, as the end products of reproductive stress, and may result in autoimmunity, chronic infections, major depression or atherosclerosis through a dysregulation of the pro/anti-inflammatory and T helper (Th) 1/Th2 cytokine balance (Calcagni & Elenkov 2006). Free glucocorticoids and catecholamines systemically induce an inhibition of cytokines produced by antigen-presenting cells and Th1 cells, like interleukin (IL)-12, interferon (IFN)-γ, IFN-α and tumor necrosis factor (TNF)-α and to an upregulation of cytokines produced by Th2 cells, such as IL-4, IL-10 and IL-13 (Elenkov & Chrousos 1999, Elenkov 2003). Moreover, high maternal cortisol levels can suppress T-cell proliferation and reduce lymphocyte sensitivity to glucocorticoids via binding on GRs (Vianna *et al.* 2011). These changes negatively affect the immune system and anti-inflammation effects throughout the process of reproductive stress.

## The important application of reproductive stress hypothesis

### Novel insights into human diseases from the reproductive stress hypothesis perspective

The reproductive stress hypothesis can be used to predict the risk of emerging maternal diseases and pathogenesis of commonly seen diseases, such as pregnancy-induced hypertension syndrome (PIH) and gestational diabetes mellitus (GDM).

#### Cushing’s syndrome during pregnancy

Prolonged hypersecretion of free cortisol can induce Cushing’s syndrome (Bronstein *et al.* 2015, Caimari *et al.* 2017). The presence of free cortisol can explain some of the maternal phenotypic changes associated with pregnancy such as light concentric obesity, moon face, fluid retention, supraclavicular fat pads (Wallace *et al.* 1996), feeling nervous and agitated, as well as antenatal and postpartum depression (Kammerer *et al.* 2006). Appreciation for this potential relationship emphasizes the importance of proper maternal care for successful health outcomes, especially for multiple pregnancies.

Various causes of CBG deficiency can aggravate Cushing's syndrome during pregnancy. The level of CBG is regulated by several relevant hormones, such as estrogen, and CBG decreases in the case of hyperthyroidism (Agbaht *et al.* 2014). Thus, there is ample reason to suspect that hyperthyroidism and impaired liver function pose an increased risk of Cushing’s syndrome onset.

#### Maternal postnatal adrenocortical hypofunction syndrome, the significance of natural labor and the risk of cesarean

Fetal CRH and cortisol, exogenous hormones for the mother, are transferred to the maternal circulatory system and induce passive reproductive stress. Because of the negative feedback loop, high levels of free cortisol inhibit the secretion of CRH and ACTH, which may result in hypothalamic–pituitary–adrenocortical hypofunction. A sudden withdraw of the exogenous hormones during labor may cause hypoadrenalism and may cause serious adrenal crisis in the pregnant woman. The stimulation of labor pain reactivates the HPA axis, which may be important for preventing the adrenal crisis and may also reduce the incidence of postpartum depression. Thus, spontaneous labor among women and animals has important clinical consequences.

Currently, cesarean section (CS) is prevalent worldwide and it is a global concern. China stands out as one of the countries with the highest rates with about 50.0% of deliveries (Lumbiganon *et al.* 2010, Wang *et al.* 2017). Concomitantly, the number and rate of multiple births have dramatically risen, mainly attributable to reproductive technology such as *in vitro* fertilization. The CS rate for multiple pregnancies has also increased, largely due to the perception that CS can improve neonatal outcomes. Based on the reproductive stress hypothesis, at least two risks associated with CS can be predicted and prevented. The first is that CS may increase the risk of postpartum depression. Secondly, multiple gestations result in severe reproductive stress, particularly passive reproductive stress, which may lead to severe hypofunction of HPA that presents as shock, coma and/or death.

#### PIH and GDM

PIH and GDM are the main contributors to adverse maternal and fetal outcomes worldwide, especially maternal and fetal death. Indeed, PIH and GDM, both important components of metabolic syndrome (Veerbeek *et al.* 2015), are driven by similar determinants (Guariguata *et al.* 2014) including biological and genetic history, depression, short stature, older maternal age and obesity or undernutrition (Werner *et al.* 2015, Dolin & Kominiarek 2018, Mizushiri *et al.* 2018). Hypertensive pregnancy disorders, particularly GDM, are closely (Leng *et al.* 2015), and possibly directly, linked with subsequent cardiovascular morbidity (Lykke *et al.* 2009). A survey of women delivering in Denmark from 1978 to 2007 showed that mothers with PIH have a 3.12-fold greater risk of type 2 diabetes (Lykke *et al.* 2009). Another study reported the PIH-related morbidity among pregnant women in Simao City, Yunnan Province of China, was 3.6%, while the morbidity among impoverished immigrant women was 57.5% due to malnutrition (Li *et al.* 2006 in Chinese). Similar results were found for women with GDM. According to the global prevalence data of GDM (aged 20–49 years) in 2013, the highest prevalence was found in Southeast Asia (25.0%) compared with 10.4% in North America and the Caribbean (Guariguata *et al.* 2014). It is estimated that about 90% of cases of GDM occur in low- and middle-income countries (Guariguata *et al.* 2014, Goldenberg *et al.* 2016).

The pathogenesis of PIH and GDM is not very clear, but the reproductive stress hypothesis may provide an explanation. Incremental levels of free CRH and cortisol can cause severe passive stress, resulting in elevated blood pressure and blood sugar during pregnancy. The passive reproductive stress is regulated by CRH-BP and fetal free cortisol. Approximately 90% of the cortisol in circulation is bound to proteins (CBG and albumin) that are inactivated (Lewis *et al.* 2005) and the remaining unbound fraction is biologically active. If the CBG is low or has impaired function and albumin is low, the free cortisol increases markedly, thereafter affecting the HPA axis (Lewis *et al.* 2005). Theoretically, factors that can lower immunoreactive CBG and albumin as well as increase free CRH and cortisol levels, such as malnutrition, negative nitrogen balance, liver diseases and kidney dysfunction during pregnancy, may lead to PIH and GDM. Consequently, the activated HPA axis and SAS should be considered as a fundamental cause of PIH and GDM, inducing the increased risk for elevated blood pressure and diabetes (O’Keeffe & St-Onge 2013, Hayase *et al.* 2014).

Numerous studies have confirmed this hypothesis. With PIH for example, maternal malnutrition, encompassing being overweight, obesity and undernutrition (especially protein restriction), affects the HPA axis by reducing the function of placental 11β-hydroxysteroid dehydrogenase type 2 enzyme (11β-HSD2). 11β-HSD2 is the fetoplacental barrier to maternal glucocorticoids, which oxidizes bioactive cortisol into bio-inactive cortisone (Salvante *et al.* 2017). This in turn increases fetal exposure to maternal cortisol, suggesting that maternal malnutrition may have an impact not only on nutritional programming, but also on fetal stress response (Micali & Treasure 2009), thus resulting in passive reproductive stress. Studies have shown that changes in 11β-HSD2 protein activity, which is expressed in the kidney, significantly affects blood pressure levels in healthy adults (Ferrari *et al.* 2001). Hypermethylation of the 11β-HSD2 promoter leads to higher levels of cortisol relative to cortisone through decreasing 11β-HSD2 synthesis, eventually facilitating the hypertensive phenotype (Ferrari *et al.* 2001, Argentieri *et al.* 2017). Meanwhile, high levels of free cortisol can affect the regulation of the kidney's sodium uptake, alongside aldosterone, and can therefore have a direct impact on salt-induced hypertension (Hunter *et al.* 2014). Accordingly, it is acceptable to presume supplementation of albumin and CRH-BP, nutritional enhancement or reducing the levels of free cortisol may prevent PIH and GDM.

Malnutrition is a broad term that includes many different manifestations of nutritional deficiencies, including undernutrition and obesity. Its main characteristic is unbalanced energy intake and consumption. It is said that many low- and middle-income countries bear the double burden of malnutrition, with stunted growth, lack of essential nutrition, along with obesity in the national population and families (Black *et al.* 2013). In the past 10 years, the nutritional health of pregnant women in China has been greatly improved, and undernutrition has been reduced, but the prevalence of relative malnutrition in pregnant women has increased. The fetus is developing rapidly over time and needs a lot of nutrients in the third trimester and may result in relative undernutrition. To meet the needs of the fetus, mothers mobilize their nutritional storage (such as calcium), which easily leads to negative nitrogen balance and aggravated stress. This may subsequently induce HIP, GDM and other complications. Especially in China in 2015, following the implementation of the two-child policy, the situation became serious because of the increasing number of advanced-age pregnant females.

### Prevention of reproductive stress syndrome

To reduce the negative effects of reproductive stress syndrome, certain measures may be taken to (1) ensure maternal nutrition supply is sufficient, especially balanced protein-energy supplementation to avoid the negative nitrogen balance during pregnancy and parturition; (2) modulate the level of CRH and free cortisol to avoid excessive stress; (3) improve the immune ability of the pregnant female and take preventative measures for osteoporosis; (4) ensure that all pregnant women have access to skilled care, including mental health services, during pregnancy, childbirth and the postpartum period; (5) use glucocorticoids with caution in the treatment of reproductive stress syndrome because of side effects that lower maternal immunity, or even worse, restrict the fetal development and growth as evidenced by reduced birth weight and impaired neuronal development reported for these fetuses (Newnham *et al.* 1999). However, a replacement of glucocorticoids may be recommended for therapy of hypoadrenalism after parturition.

## Conclusions

In summary, we presented the reproductive stress hypothesis describing the physiological responses to stress in pregnant females during reproductive events. Reproductive stress is a double-edged sword. On the one hand, appropriate reproductive stress is a prerequisite for performing successful reproductive processes. On the other hand, inadequate or excessive reproductive stress may impair reproductive functions and result in a number of complications. The reproductive stress hypothesis is helpful to account for the onset of parturition and to predict some diseases using a novel framework. Necessary interventions should be taken for preventing the negative consequences of reproductive stress syndrome.

## Declaration of interest

The authors declare that there is no conflict of interest that could be perceived as prejudicing the impartiality of this review.

## Funding

This work was supported in part by the National Key Research and Development Program of China (No. 2016YFD0501209) and Hunan Provincial Natural Science Foundation of China (No. 2017JJ3108).

## References

[bib1] Agbaht K Gullu S 2014 Adrenocortical reserves in hyperthyroidism. Endocrine 136–143. (10.1007/s12020-013-9933-y) 23532634

[bib2] Altemus M Roca C Galliven E Romanos C Deuster P 2001 Increased vaopressin and adrenocorticotropin responses to stress in the midluteal phase of the menstrual cycle. Journal of Clinical Endocrinology and Metabolism 2525–2530. (10.1210/jcem.86.6.7596) 11397850

[CIT107] Argentieri MA Nagarajan S Seddighzadeh B Baccarelli AA Shields AE 2017 Epigenetic Pathways in Human Disease: The Impact of DNA Methylation on Stress-Related Pathogenesis and Current Challenges in Biomarker Development. EBioMedicine 18 327–350. (10.1016/j.ebiom.2017.03.044) 28434943 PMC5405197

[bib3] Atkinson HC Waddell BJ 1997 Circadian variation in basal plasma corticosterone and adrenocorticotropin in the rat: sexual dimorphism and changes across the estrous cycle. Endocrinology 3842–3848. (10.1210/endo.138.9.5395) 9275073

[bib4] August P Mueller FB Sealey JE Edersheim TG 1995 Role of renin-angiotensin system in blood pressure regulation in pregnancy. Lancet 896–897. (10.1016/s0140-6736(95)90012-8) 7707813

[bib5] Baan M Taverne MA de Gier J Kooistra HS Kindahl H Dieleman SJ Okkens AC 2008 Hormonal changes in spontaneous and aglepristone-induced parturition in dogs. Theriogenology 399–407. (10.1016/j.theriogenology.2007.10.008) 18054071

[bib6] Ben-Jonathan N Maxson RE 1978 Elevation of dopamine in fetal plasma and the amniotic fluid during gestation. Endocrinology 649–652. (10.1210/endo-102-2-649) 743985

[bib7] Black RE Victora CG Walker SP Bhutta ZA Christian P de Onis M Ezzati M Grantham-McGregor S Katz J Martorell R ***et al***. 2013 Maternal and child undernutrition and overweight in low-income and middle-income countries. Lancet 427–451. (10.1016/S0140-6736(13)60937-X) 23746772

[bib8] Bronstein MD Machado MC Fragoso MCBV 2015 Management of endocrine disease: management of the pregnant patient with Cushings. European Journal of Endocrinology R85–R91. (10.1530/EJE-14-1130) 25872515

[bib9] Brownfoot FC Gagliardi DI Bain E Middleton P Crowther CA 2013 Different corticosteroids and regimens for accelerating fetal lung maturation for women at risk of preterm birth. Cochrane Database of Systematic Reviews CD006764. (10.1002/14651858.CD006764.pub3) 23990333

[bib10] Brue T Amodru V Castinetti F 2018 MANAGEMENT of ENDOCRINE DISEASE: Management of Cushing’s syndrome during pregnancy: solved and unsolved questions. European Journal of Endocrinology R259–R266. (10.1530/EJE-17-1058) 29523633

[bib11] Busada JT Cidlowski JA 2017 Mechanisms of glucocorticoid action during development. Nuclear Receptors in Development and Disease 147–170. (10.1016/bs.ctdb.2016.12.004) 28527570

[bib13] Buss C Davis EP Shahbaba B Pruessner JC Head K Sandman CA 2012 Maternal cortisol over the course of pregnancy and subsequent child amygdala and hippocampus volumes and affective problems. PNAS E1312–E1319. (10.1073/pnas.1201295109) 22529357 PMC3356611

[bib15] Caimari F Valassi E Garbayo P Steffensen C Santos A Corcoy R Webb SM 2017 Cushing’s syndrome and pregnancy outcomes: a systematic review of published cases. Endocrine 555–563. (10.1007/s12020-016-1117-0) 27704478

[bib14] Calcagni E Elenkov I 2006 Stress system activity, innate and T helper cytokines, and susceptibility to immune-related diseases. Annals of the New York Academy of Sciences 62–76. (10.1196/annals.1351.006) 16855135

[bib16] Carlsen SM Jacobsen G Bjerve KS 2003 Androgen levels in pregnant women decrease with increasing maternal age. Scandinavian Journal of Clinical and Laboratory Investigation 23–26. (10.1080/00365510310000457) 12729066

[bib17] Carter JR Goldstein DS 2015 Sympathoneural and adrenomedullary responses to mental stress. Comprehensive Physiology 119–146. (10.1002/cphy.c140030) 25589266 PMC5280073

[bib18] Challis JR Lye SJ Gibb W 1997 Prostaglandins and parturition. Annals of the New York Academy of Sciences 254–267. (10.1111/j.1749-6632.1997.tb48546.x) 9329846

[bib19] Charkoudian N Usselman CW Skow RJ Staab JS Julian CG Stickland MK Chari RS Khurana R Davidge ST Davenport MH ***et al***. 2017 Muscle sympathetic nerve activity and volume-regulating factors in healthy pregnant and nonpregnant women. American Journal of Physiology: Heart and Circulatory Physiology H782–H787. (10.1152/ajpheart.00312.2017) 28733450 PMC6148088

[bib20] Chen Y Holzman C Chung H Senagore P Talge NM Siler-Khodr T 2010 Levels of maternal serum corticotropin-releasing hormone (CRH) at midpregnancy in relation to maternal characteristics. Psychoneuroendocrinology 820–832. (10.1016/j.psyneuen.2009.11.007) 20006448 PMC2875356

[bib21] Christian LM 2012 Physiological reactivity to psychological stress in human pregnancy: current knowledge and future directions. Progress in Neurobiology 106–116. (10.1016/j.pneurobio.2012.07.003) 22800930 PMC3479316

[bib22] Chrousos GP 2000 The role of stress and the hypothalamic-pituitary-adrenal axis in the pathogenesis of the metabolic syndrome: neuroendocrine and target tissue-related causes. Internation Journal of Obesity and Related Metabolism Disordors S50–S55. 10.1038/sj.ijo.0801278 10997609

[bib24] Ciechanowska M Kowalczyk M Lapot M Malewski T Antkowiak B Brytan M Winnicka I Przekop F 2018 Effect of corticotropin releasing hormone and corticotropin releasing hormone antagonist on biosynthesis of gonadotropin relasing hormone and gonadotropin relasing hormone receptor in the hypothalamic-pituitary unit of follicular-phase ewes and contribution of kisspeptin. Journal of Physiology and Pharmacology 451–461. (10.26402/jpp.2018.3.13) 30342430

[bib25] Dobson H Ghuman S Prabhakar S Smith R 2003 A conceptual model of the influence of stress on female reproduction. Reproduction 151–163. (10.1530/rep.0.1250151) 12578529

[bib26] Dolin CD Kominiarek MA 2018 Pregnancy in women with obesity. Obstetrics and Gynecology Clinics of North America 217–232. (10.1016/j.ogc.2018.01.005) 29747727

[bib27] Eisman J 1998 Relevance of pregnancy and lactation to osteoporosis? Lancet 504–505. (10.1016/S0140-6736(05)79245-X) 9716051

[bib29] Elenkov IJ 2004 Glucocorticoids and the th1/th2 balance. Annals of the New York Academy of Sciences 1024 138–146. (10.1196/annals.1321.010) 15265778

[bib28] Elenkov IJ Chrousos GP 1999 Stress hormones, Th1/Th2 patterns, pro/anti-inflammatory cytokines and susceptibility to disease. Trends in Endocrinology and Metabolism 359–368. (10.1016/S1043-2760(99)00188-5) 10511695

[bib30] Ferrari P Sansonnens A Dick B Frey FJ 2001 In vivo 11beta-HSD-2 activity: variability, salt-sensitivity, and effect of licorice. Hypertension 1330–1336. (10.1161/hy1101.096112) 11751713

[bib31] Fowden AL Forhead AJ 2011 Adrenal glands are essential for activation of glucogenesis during undernutrition in fetal sheep near term. American Journal of Physiology: Endocrinology and Metabolism E94–E102. (10.1152/ajpendo.00205.2010) 20959526 PMC3023201

[bib32] Gilles M Otto H Wolf IAC Scharnholz B Peus V Schredl M Sütterlin MW Witt SH Rietschel M Laucht M ***et al***. 2018 Maternal hypothalamus-pituitary-adrenal (HPA) system activity and stress during pregnancy: effects on gestational age and infant's anthropometric measures at birth. Psychoneuroendocrinology 152–161. (10.1016/j.psyneuen.2018.04.022) 29783163

[bib33] Goldenberg RL McClure EM Harrison MS Miodovnik M 2016 Diabetes during pregnancy in low- and middle-income countries. American Journal of Perinatology 1227–1235. (10.1055/s-0036-1584152) 27182990

[bib34] Gore AC Attardi B DeFranco DB 2006 Glucocorticoid repression of the reproductive axis: effects on GnRH and gonadotropin subunit mRNA levels. Molecular and Cellular Endocrinology 40–48. (10.1016/j.mce.2006.06.002) 16839661

[bib35] Grachev P Li XF Hu MH Li SY Millar RP Lightman SL O'Byrne KT 2014 Neurokinin b signaling in the female rat: a novel link between stress and reproduction. Endocrinology 2589–2601. (10.1210/en.2013-2038) 24708241

[bib36] Guariguata L Linnenkamp U Beagley J Whiting DR Cho NH 2014 Global estimates of the prevalence of hyperglycaemia in pregnancy. Diabetes Research and Clinical Practice 176–185. (10.1016/j.diabres.2013.11.003) 24300020

[bib37] Hacker AN Fung EB King JC 2012 Role of calcium during pregnancy: maternal and fetal needs. Nutrition Reviews 397–409. (10.1111/j.1753-4887.2012.00491.x) 22747842

[bib39] Harp JB Yancopoulos GD Gromada J 2016 Glucagon orchestrates stress-induced hyperglycaemia. Diabetes, Obesity and Metabolism 648–653. (10.1111/dom.12668) PMC5084782 27027662

[bib38] Hayase M Shimada M Seki H 2014 Sleep quality and stress in women with pregnancy-induced hypertension and gestationaldiabetes mellitus. Women and Birth 190–195. (10.1016/j.wombi.2014.04.002) 24881523

[bib40] Heino A Gissler M Hindori-Mohangoo AD Blondel B Klungsøyr K Verdenik I Mierzejewska E Velebil P Sól Ólafsdóttir H Macfarlane A ***et al***. 2016 Variations in multiple birth rates and impact on perinatal outcomes in Europe. PLoS ONE e0149252. (10.1371/journal.pone.0149252) 26930069 PMC4773186

[bib42] Hobel CJ Goldstein A Barrett ES 2008 Psychosocial stress and pregnancy outcome. Clinical Obstetrics and Gynecology 333–348. (10.1097/GRF.0b013e31816f2709) 18463464

[bib43] Hunter RW Ivy JR Bailey MA 2014 Glucocorticoids and renal Na^+^ transport: implications for hypertension and salt sensitivity. Journal of Physiology 1731–1744. (10.1113/jphysiol.2013.267609) 24535442 PMC4001748

[bib41] Hydbring E Madej A MacDonald E Drugge-Boholm G Berglund B Olsson K 1999 Hormonal changes during parturition in heifers and goats are related to the phases and severity of labour. Journal of Endocrinology 75–85. (10.1677/joe.0.1600075) 9854179

[bib44] Jensen E Wood C Keller-Wood M 2002 The normal increase in adrenal secretion during pregnancy contributes to maternal volume expansion and fetal homeostasis. Journal of the Society for Gynecologic Investigation 362–371. (10.1177/107155760200900607) 12445601

[bib45] Jensen E Wood CE Keller-Wood M 2005 Chronic alterations in ovine maternal corticosteroid levels influence uterine blood flow and placental and fetal growth. American Journal of Physiology: Regulatory, Integrative and Comparative Physiology R54–R61. (10.1152/ajpregu.00149.2004) 15231491

[bib46] Jung C Ho JT Torpy DJ Rogers A Doogue M Lewis JG Czajko RJ Inder WJ 2011 A longitudinal study of plasma and urinary cortisol in pregnancy and postpartum. Journal of Clinical Endocrinology and Metabolism 1533–1540. (10.1210/jc.2010-2395) 21367926

[bib47] Jwa SC Fujiwara T Yamanobe Y Kozuka K Sago H 2015 Changes in maternal hemoglobin during pregnancy and birth outcomes. BMC Pregnancy and Childbirth 80. (10.1186/s12884-015-0516-1) 25884586 PMC4389317

[bib48] Kamba A Daimon M Murakami H Otaka H Matsuki K Sato E Tanabe J Takayasu S Matsuhashi Y Yanagimachi M ***et al***. 2016 Association between higher serum cortisol levels and decreased insulin secretion in a GeneralPopulation. PLoS ONE e0166077. (10.1371/journal.pone.0166077) 27861636 PMC5115704

[bib49] Kammerer M Taylor A Glover V 2006 The HPA axis and perinatal depression: a hypothesis. Archives of Women’s Mental Health 187–196. (10.1007/s00737-006-0131-2) 16708167

[bib50] Khajehei M Doherty M Tilley PJM Sauer K 2015 Prevalence and risk factors of sexual dysfunction in postpartum Australian women. Journal of Sexual Medicine 1415–1426. (10.1111/jsm.12901) 25963126

[bib51] Komori T 2016 Glucocorticoid signaling and bone biology. Hormone and Metabolic Research 755–763. (10.1055/s-0042-110571) 27871116

[bib52] Kovacs CS Ralston SH 2015 Presentation and management of osteoporosis presenting in association with pregnancy or lactation. Osteoporosis International 2223–2241. (10.1007/s00198-015-3149-3) 25939309

[bib53] Lebenthal A Lebenthal E 1999 The ontogeny of the small intestinal epithelium. Journal of Parenteral and Enteral Nutrition S3–S6. (10.1177/014860719902300502) 10483884

[bib54] Leng J Shao P Zhang C Tian H Zhang F Zhang S Dong L Li L Yu Z Chan JC ***et al***. 2015 Prevalence of gestational diabetes mellitus and its risk factors in Chinese pregnant women: a prospective population-based study in Tianjin, China. PLoS ONE e0121029. (10.1371/journal.pone.0121029) 25799433 PMC4370728

[bib55] Lewis JG Bagley CJ Elder PA Bachmann AW Torpy DJ 2005 Plasma free cortisol fraction reflects levels of functioning corticosteroid-binding globulin. Clinica Chimica Acta: International Journal of Clinical Chemistry 189–194. (10.1016/j.cccn.2005.03.044) 15904907

[bib56] Li X Duan L Xiao H 2006 思茅市贫困移民妇女妊娠高血压综合征病因分析. Harbin Medical Journal 24.

[bib57] Lucy MC 2008 Functional differences in the growth hormone and insulin-like growth factor axis in cattle and pigs: implications for postpartum nutrition and reproduction. Reproduction in Domestic Animals 31–39. (10.1111/j.1439-0531.2008.01140.x) 18638098

[bib58] Lumbers ER Pringle KG 2014 Roles of the circulating renin-angiotensin-aldosterone system in human pregnancy. American Journal of Physiology: Regulatory, Integrative and Comparative Physiology R91–101. (10.1152/ajpregu.00034.2013) 24089380

[bib59] Lumbiganon P Laopaiboon M Gulmezoglu AM Souza JP Taneepanichskul S Ruyan P Attygalle DE Shrestha N Mori R Nguyen DH ***et al***. 2010 Method of delivery and pregnancy outcomes in Asia: the WHO global survey on maternal and perinatal health 2007-08. Lancet 490–499. (10.1016/S0140-6736(09)61870-5) 20071021

[bib60] Lykke JA Langhoff-Roos J Sibai BM Funai EF Triche EW Paidas MJ 2009 Hypertensive pregnancy disorders and subsequent morbidity and type 2 diabetesmellitus in the mother. Hypertension 944–951. (10.1161/HYPERTENSIONAHA.109.130765) 19433776

[bib61] Maršál K 2018 Physiological adaptation of the growth-restricted fetus. Best Practice and Research: Clinical Obstetrics and Gynaecology 37–52. (10.1016/j.bpobgyn.2018.02.006) 29753694

[bib62] Mastorakos G Ilias I 2000 Maternal hypothalamic-pituitary-adrenal axis in pregnancy and the postpartum period. Postpartum-related disorders. Annals of the New York Academy of Sciences 95–106. (10.1111/j.1749-6632.2000.tb06220.x) 10818396

[bib63] Mastorakos G Ilias I 2003 Maternal and fetal hypothalamic-pituitary-adrenal axes during pregnancy and postpartum. Annals of the New York Academy of Sciences 136–149. (10.1196/annals.1290.016) 14644820

[bib65] McLean M Smith R 1999 Corticotropin-releasing hormone in human pregnancy and parturition. Trends in Endocrinology and Metabolism 174–178. (10.1016/S1043-2760(98)00146-5) 10370225

[bib64] McLean M Bisits A Davies J Woods R Lowry P Smith R 1995 A placental clock controlling the length of human pregnancy. Nature Medicine 460–463. (10.1038/nm0595-460) 7585095

[bib67] Menon R Bonney EA Condon J Mesiano S Taylor RN 2016 Novel concepts on pregnancy clocks and alarms: redundancy and synergy in human parturition. Human Reproduction Update 535–560. (10.1093/humupd/dmw022) 27363410 PMC5001499

[bib71] Micali N Treasure J 2009 Biological effects of a maternal ED on pregnancy and foetal development: a review. European Eating Disordors Review 448–454. (10.1002/erv.963) 19851992

[bib68] Miranda A Sousa N 2018 Maternal hormonal milieu influence on fetal brain development. Brain and Behavior e00920. (10.1002/brb3.920) 29484271 PMC5822586

[bib70] Mitchell BF Taggart MJ 2009 Are animal models relevant to key aspects of human parturition? American Journal of Physiology: Regulatory, Integrative and Comparative Physiology R525–R545. (10.1152/ajpregu.00153.2009) 19515978

[bib69] Mitchell JC Li XF Breen L Thalabard JC O'Byrne KT 2005 The role of the locus coeruleus in corticotropin-releasing hormone and stress-induced suppression of pulsatile luteinizing hormone secretion in the female rat. Endocrinology 323–331. (10.1210/en.2004-1053) 15486230

[bib72] Mizushiri S Daimon M Murakami H Kamba A Osonoi S Yamaichi M Matsumura K Tanabe J Matsuhashi Y Yanagimachi M ***et al***. 2018 Lower serum calcium levels are a risk factor for a decrease in eGFR in a general non-chronic kidney disease population. Scientific Reports 14213. (10.1038/s41598-018-32627-4) 30242201 PMC6155105

[bib73] Newnham JP Evans SF Godfrey M Huang W Ikegami M Jobe A 1999 Maternal, but not fetal, administration of corticosteroids restricts fetal growth. Journal of Maternal-Fetal Medicine 81–87. (10.1002/(SICI)1520-6661(199905/06)8:3<81::AID-MFM3>3.0.CO;2-N) 10338060

[bib74] Novy MJ 1977 Endocrine and pharmacological factors which influence the onset of labour in rhesus monkeys. Ciba Foundation Symposium 259–295. (10.1002/9780470720295.ch11) 205394

[bib75] O’Keeffe M St-Onge MP 2013 Sleep duration and disorders in pregnancy: implications for glucose metabolism and pregnancy outcomes. International Journal of Obesity 765–770. (10.1038/ijo.2012.142) 22945608 PMC3836666

[bib76] Oyola MG Handa RJ 2017 Hypothalamic-pituitary-adrenal and hypothalamic-pituitary-gonadal axes: sex differences in regulation of stress responsivity. Stress 476–494. (10.1080/10253890.2017.1369523) 28859530 PMC5815295

[bib77] Papageorghiou AT Avgidou K Bakoulas V Sebire NJ Nicolaides KH 2006 Risks of miscarriage and early preterm birth in trichorionic triplet pregnancies with embryo reduction versus expectant management: new data and systematic review. Human Reproduction 1912–1917. (10.1093/humrep/del048) 16613889

[bib79] Parisi F di Bartolo I Savasi VM Cetin I 2019 Micronutrient supplementation in pregnancy: wo, what and how much? Obstetric Medicine 5–13. (10.1177/1753495X18769213) PMC6416688 30891086

[bib78] Power ML Bowman ME Smith R Ziegler TE Layne DG Schulkin J Tardif SD 2006 Pattern of maternal serum corticotropin-releasing hormone concentration during pregnancy in the common marmoset (Callithrix jacchus). American Journal of Primatology 181–188. (10.1002/ajp.20215) 16429419

[bib80] Racicot K Kwon JY Aldo P Silasi M Mor G 2014 Understanding the complexity of the immune system during pregnancy. American Journal of Reproductive Immunology 107–116. (10.1111/aji.12289) 24995526 PMC6800182

[bib81] Raftogianni A Roth LC García-González D Bus T Kühne C Monyer H Spergel DJ Deussing JM Grinevich V 2018 Deciphering the contributions of CRH receptors in the brain and pituitary to stress-induced inhibition of the reproductive axis. Frontiers in Molecular Neuroscience 305. (10.3389/fnmol.2018.00305) 30214395 PMC6125327

[bib82] Ravanos K Dagklis T Petousis S Margioula-Siarkou C Prapas Y Prapas N 2015 Factors implicated in the initiation of human parturition in term and preterm labor: a review. Gynecological Endocrinology 679–683. (10.3109/09513590.2015.1076783) 26303116

[bib83] Rog-Zielinska EA Richardson RV Denvir MA Chapman KE 2014 Glucocorticoids and foetal heart maturation; implications for prematurity and foetal programming. Journal of Molecular Endocrinology R125–R135. (10.1530/JME-13-0204) 24299741

[bib84] Russell JA Douglas AJ Brunton PJ 2008 Reduced hypothalamo-pituitary-adrenal axis stress responses in late pregnancy: central opioid inhibition and noradrenergic mechanisms. Annals of the New York Academy of Sciences 428–438. (10.1196/annals.1410.032) 19120138

[bib85] Salvante KG Milano K Kliman HJ Nepomnaschy PA 2017 Placental 11 β-hydroxysteroid dehydrogenase type 2 (11β-HSD2) expression very early during human pregnancy. Journal of Developmental Origins of Health and Disease 149–154. (10.1017/S2040174416000611) 28112069

[bib86] Saudek CD Finkowski M Knopp RH 1975 Plasma glucagon and insulin in rat pregnancy. Roles in glucose homeostasis. Journal of Clinical Investigation 180–187. (10.1172/JCI107909) 1109177 PMC301730

[bib87] Schneiderman M Czuzoj-Shulman N Spence AR Abenhaim HA 2017 Maternal and neonatal outcomes of pregnancies in women with Addison’s disease: a population-based cohort study on 7.7 million births. BJOG 1772–1779. (10.1111/1471-0528.14448) 27981742

[bib88] Smith SM Vale WW 2006 The role of the hypothalamic-pituitary-adrenal axis in neuroendocrine responses to stress. Dialogues in Clinical Neuroscience 383–395. 17290797 10.31887/DCNS.2006.8.4/ssmith PMC3181830

[bib92] Sorem KA Smikle CB Spencer DK Yoder BA Graveson MA Siler-Khodr TM 1996 Circulating maternal corticotropin-releasing hormone and gonadotropin-releasing hormone in normal and abnormal pregnancies. American Journal of Obstetrics and Gynecology 912–916. (10.1016/s0002-9378(96)80024-x) 8885747

[bib93] Thomson M 1998 Does the CRH binding protein shield the anterior pituitary from placental CRH? Endocrine 221–226. (10.1385/ENDO:9:3:221) 10221586

[bib94] Tombeau Cost K Unternaehrer E Plamondon A Steiner M Meaney M Atkinson L Kennedy JL Fleming AS & MAVAN Research Team 2017 Thinking and doing: the effects of dopamine and oxytocin genes and executive function on mothering behaviours. Genes, Brain, and Behavior 285–295. (10.1111/gbb.12337) 27620964

[bib95] Unno N Wong CH Jenkins SL Wentworth RA Ding XY Li C Robertson SS Smotherman WP Nathanielsz PW 1999 Blood pressure and heart rate in the ovine fetus: ontogenic changes and effects of fetal adrenalectomy. American Journal of Physiology H248–H256. (10.1152/ajpheart.1999.276.1.H248) 9887039

[bib96] Valsamakis G Chrousos G Mastorakos G 2019 Stress, female reproduction and pregnancy. Psychoneuroendocrinology 48–57. (10.1016/j.psyneuen.2018.09.031) 30291988

[bib97] Valsamakis G Papatheodorou DC Chalarakis N Vrachnis N Sidiropoulou EJ Manolikaki M Mantzou A Margeli A Papassotiriou I Chrousos GP ***et al***. 2017 In pregnancy increased maternal STAI trait stress score shows decreased insulin sensitivity and increased stress hormones. Psychoneuroendocrinology 11–16. (10.1016/j.psyneuen.2017.06.008) 28647674

[bib98] Van de Kar LD Blair ML 1999 Forebrain pathways mediating stress induced hormone secretion. Frontiers in Neuroendocrinology 1–48. (10.1006/frne.1998.0172) 9882535

[bib99] Veerbeek JH Hermes W Breimer AY van Rijn BB Koenen SV Franx A de Groot CJ Koster MP 2015 Cardiovascular disease risk factors after early-onset preeclampsia, late-onset preeclampsia, and pregnancy-induced hypertension. Hypertension 600–606. (10.1161/HYPERTENSIONAHA.114.04850) 25561694

[bib100] Verdonk K Visser W Van Den Meiracker AH Danser AH 2014 The renin-angiotensin-aldosterone system in pre-eclampsia: the delicate balance between good and bad. Clinical Science 537–544. (10.1042/CS20130455) 24400721

[bib101] Vianna P Bauer ME Dornfeld D Chies JA 2011 Distress conditions during pregnancy may lead to pre-eclampsia by increasing cortisol levels and altering lymphocyte sensitivity to glucocorticoids. Medical Hypotheses 188–191. (10.1016/j.mehy.2011.04.007) 21550175

[bib102] Viau V Meaney MJ 1991 Variations in the hypothalamic-pituitary-adrenal response to stress during the estrous cycle in the rat. Endocrinology 2503–2511. (10.1210/endo-129-5-2503) 1657578

[CIT109] Vogel J P Chawanpaiboon S Moller A-B Watananirun K Boneta M Lumbiganon P 2018 The global epidemiology of preterm birth. Best Practice & Research Clinical Obstetrics & Gynaecology 52 3–12. (10.1016/j.bpobgyn.2018.04.003) 29779863

[bib103] Wagenmaker ER Moenter SM 2017 Exposure to acute psychosocial stress disrupts the luteinizing hormone surge independent of estrous cycle alterations in female mice. Endocrinology 2593–2602. (10.1210/en.2017-00341) 28549157 PMC5551545

[bib104] Wallace C Toth EL Lewanczuk RZ Siminoski K 1996 Pregnancy-induced Cushing’s syndrome in multiple pregnancies. Journal of Clinical Endocrinology and Metabolism 15–21. (10.1210/jcem.81.1.8550743) 8550743

[bib105] Walsh SW Williams EJ Evans AC 2011 A review of the causes of poor fertility in high milk producing dairy cows. Animal Reproduction Science 127–138. (10.1016/j.anireprosci.2010.12.001) PMC7125520 21255947

[bib106] Wang X Hellerstein S Hou L Zou L Ruan Y Zhang W 2017 Caesarean deliveries in China. BMC Pregnancy and Childbirth 54. (10.1186/s12884-017-1233-8) 28166782 PMC5294866

[bib107] Weinstein RS Wan C Liu Q Wang Y Almeida M O'Brien CA Thostenson J Roberson PK Boskey AL Clemens TL ***et al***. 2010 Endogenous glucocorticoids decrease skeletal angiogenesis, vascularity, hydration, and strength in aged mice. Aging Cell 147–161. (10.1111/j.1474-9726.2009.00545.x) 20047574 PMC2858771

[bib108] Werner EF Braun JM Yolton K Khoury JC Lanphear BP 2015 The association between maternal urinary phthalate concentrations and blood pressure in pregnancy: the HOME Study. Environmental Health 75. (10.1186/s12940-015-0062-3) 26380974 PMC4574131

[bib109] West CA Han W Li N Masilamani SME 2014 Renal epithelial sodium channel is critical for blood pressure maintenance and sodium balance in the normal late pregnant rat. Experimental Physiology 816–823. (10.1113/expphysiol.2013.076273) 24563165

[bib110] Whirledge SD Oakley RH Myers PH Lydon JP DeMayo F Cidlowski JA 2015 Uterine glucocorticoid receptors are critical for fertility in mice through control of embryo implantation and decidualization. PNAS 15166–15171. (10.1073/pnas.1508056112) 26598666 PMC4679013

[bib111] Whittaker PG Macphail S Lind T 1996 Serial hematologic changes and pregnancy outcome. Obstetrics and Gynecology 33–39. (10.1016/0029-7844(96)00095-6) 8684758

[bib112] Wood CE Walker CD 2015 Fetal and neonatal HPA axis. Comprehensive Physiology 33–62. (10.1002/cphy.c150005) 26756626

[CIT108] Zeitlin J Szamotulska K Drewniak N Mohangoo AD Chalmers J Sakkeus L Irgens L Gatt M Gissler M Blondel B ***et al***. 2013 Preterm birth time trends in Europe: a study of 19 countries. BJOG-An International Journal of Obstetrics and Gynaecology 1356–1365. (10.1111/1471-0528.12281) 23700966 PMC4285908

